# Association between global biomarker of oxidative stress and quantitative ultrasound parameters in middle-aged and elderly adults: A cross-sectional study

**DOI:** 10.3389/fpubh.2022.1032550

**Published:** 2023-01-06

**Authors:** Xue Shen, Yawen Liu, Qianqian Zhao, Haitao Cheng, Binbin Li, Ann M. Vuong, Yiliang Fan, Mengmeng Zhang, Shuman Yang

**Affiliations:** ^1^Department of Epidemiology and Biostatistics, School of Public Health, Jilin University, Changchun, China; ^2^FAW General Hospital of Jilin Province, Changchun, China; ^3^Department of Epidemiology and Biostatistics, School of Public Health, University of Nevada, Las Vegas, NV, United States

**Keywords:** oxidative stress, fluorescent oxidation products, quantitative ultrasound parameters, speed of sound, broadband ultrasound attenuation, middle-aged and elderly adults

## Abstract

**Introduction:**

With the population aging, osteoporosis has become a major public health concern. Elevated oxidative stress is a vital detrimental factor for bone health. Compared to common oxidative stress-related biomarkers, Fluorescent Oxidation Products (FlOPs) reflect the global levels of oxidation from proteins, lipids, and DNA. Nevertheless, whether plasma FlOP levels are related to bone health measured by Quantitative ultrasound (QUS) is unclear. Thus, the present study examined the association between FlOPs and QUS parameters in middle-aged and elderly adults.

**Methods:**

This community-based cross-sectional study was conducted in Changchun, northeast China. Plasma FlOPs were determined by a fluorescent microplate reader at a wavelength of 320/420 nm (excitation/emission). QUS parameters [speed of sound (SOS) and broadband ultrasound attenuation (BUA)] of the calcaneus were assessed by an ultrasound bone densitometer. We used multivariable linear regression to examine the association between FlOPs and QUS parameters.

**Results:**

A total of 491 subjects were included in this study. Their average age was 65.2 years (standard deviation [SD]: 9.7 years). FlOPs were inversely associated with SOS (β for an increase of logarithmic interquartile range = −10.64; *P* = 0.018). Higher FlOP levels were marginally associated with lower SOS in females (β for an increase of logarithmic interquartile range = −9.68, *P* = 0.066), but not in males (β for an increase of logarithmic interquartile range = −11.84, *P* = 0.131). No significant relationship between FlOPs and BUA was observed.

**Conclusions:**

Plasma FlOP levels were inversely associated with SOS, but not with BUA in middle-aged and elderly adults.

## Introduction

With the aging of the population, osteoporosis has become a major public health concern, leading to lower quality of life and imposing a huge disease burden on patients, their families, and society (1, 2). In China, the prevalence of osteoporosis was 19.2% for people aged 50 years or older, and 32.0% for those over 65 years of age (3). By 2050, it is estimated that the annual number of fragility fractures in China will increase to 5.99 million and the related medical cost will reach $25.43 billion (4). In communities, usage of the quantitative ultrasound device has become a relatively convenient tool for osteoporosis screening (5).

Oxidative stress occurs from the imbalance between the production of free radicals and the antioxidant defense system. The accumulation of excessive reactive oxygen species (ROS) induces oxidative damage to tissue and cellular macromolecules (e.g., protein, lipids, and DNA), leading to increased risk of many diseases such as cardiovascular disease, chronic kidney disease, and osteoporosis (6, 7).

*In vivo* and *in vitro* evidence suggests that ROS are involved in the pathogenesis of bone loss, increasing the level of bone resorption by stimulating the differentiation of osteoclasts and reducing bone formation *via* inhibiting the activity of osteoblasts (8–10). Previous studies on the relationship between oxidative stress and bone health are primarily based on the bone mineral density (BMD) determined by dual-energy X-ray absorptiometry (DXA) (11–13), only a few surveys are based on quantitative ultrasound (QUS) parameters (14, 15). Compared to DXA, QUS is less expensive, portable, and free from ionizing radiation. Several human studies have examined the relationship between traditional oxidative stress-related biomarkers [i.e., malondialdehyde (MDA)] and bone health assessed by BMD or QUS parameters, however, their results were inconsistent (11, 12, 14, 15). A recent study conducted in Iraq suggested that MDA is negatively associated with BMD in postmenopausal women (13). In contrast, Wu et al. observed an insignificant relationship between MDA and BMD in Chinese postmenopausal women (12). This inconsistency is likely due to the fact that traditional oxidative stress biomarkers only capture one specific aspect of oxidative damage. For example, MDA and 8-hydroxyguanosine (8-OHdG) were commonly used to assess the level of lipid peroxidation and DNA oxidation, respectively (16).

Fluorescent Oxidation Products (FlOPs) reflect oxidative damage in terms of protein, lipids, DNA, and carbohydrate, and have been used as a global biomarker of oxidative stress in epidemiologic studies (17, 18). Compared to specific oxidation measurements such as MDA assay *via* colorimetric thiobarbituric acid, the fluorescence method is 10–100 times more sensitive (19). In our previous study, higher FlOP levels were found to be associated with lower hip BMD in male veterans (20) and an increased risk of hip fracture in postmenopausal women (21). However, whether FlOPs are associated with bone health determined by QUS parameters is unclear. Given the above evidence, we hypothesized that FlOPs are negatively associated with QUS parameters. QUS measurements at the calcaneal could provide information on the structural and mechanical properties of the bone (22, 23). Knowing this relationship would expand our understanding of the impact of oxidative stress on the structural and mechanical properties of bone assessed by QUS.

## Materials and methods

### Study design and participants

This study is part of the national project entitled “The Comprehensive Demonstration Research Project of Major Chronic Disease Prevention and Control Technology in Northeast China (Health Northeast)” initiated by China Medical University. As one of the important participating units, Jilin University undertook the main work of the project in Jilin. The design and implementation of the study have been previously described (24). In brief, a community-based cross-sectional study was performed between January and December 2019 in Changchun, Jilin, China. We cooperated with more than 20 community health service centers distributed in 10 districts in Changchun. All participants were interviewed face-to-face at the community health service centers using a structured questionnaire. We collected data such as socio-demographic information, medical history, and lifestyle factors. We additionally measured data such as height, weight, fasting blood glucose, and blood pressure. Because we were limited to only one QUS device, we randomly selected two community health service centers from two urban districts in Changchun for the present study. All subjects provided written informed consent. This project was approved by the institutional review board (IRB) of China Medical University.

We included all individuals aged 40 years or older, with complete and valid information on QUS parameters, who also provided blood samples. Individuals who met any of the following criteria were excluded: (1) having osteoporosis-related medications, such as bisphosphonates, estrogen, and glucocorticoids; (2) having diseases that may contribute to secondary osteoporosis (e.g., thyroid/parathyroid disorders, type 1 diabetes mellitus, chronic liver/kidney disease, rheumatoid arthritis, or cancer); and (3) having missing data on covariates, such as age, sex, and body mass index (BMI).

### Quantitative ultrasound measurement

QUS measurements of the calcaneus were performed using an ultrasound bone densitometer (Osteo KJ3000, Kejin Inc., Nanjing, Jiangsu, China). This device produces two key parameters: speed of sound (SOS, expressed as m/s) and broadband ultrasound attenuation (BUA, expressed as dB/MHz). SOS is the transmission time of sound waves divided by the length of the body part studied; BUA refers to the slope between the attenuation of sound signals and their frequency (23). The daily performed control spine phantom had a coefficient of variation (CV) of <5%. Participants with invalid QUS measurements (i.e., negative values) were excluded.

### Blood collection

Fasting blood samples (≥8 h) were collected with anticoagulant tubes (BD, Becton, Dickinson and Company, Franklin Lakes, New Jersey, USA) from all subjects. Within 4 h, these samples were transported to the laboratory of Jilin University using ice boxes for processing and stored at −80°C until assay.

### FlOP measurement

We measured FlOPs for all individuals with collected blood samples. Measurement of FlOPs was performed based on the method modified by Shimasaki (25) and Wu (26), and has been previously described (20). In brief, plasma was extracted with ethanol/ether (3:1, v/v) and centrifuged at 3,000 rpm for 10 min at 4°C. Then the supernatant was added to a black 96-well Microplate (Black Fat Bottom Polystyrene High Bind Microplate 3925, Corning) and measured by a fluorescent microplate reader (Cytation 3 Cell Imaging Multi-Mode Reader, BioTek, Vermont, USA) at a wavelength of 320/420 nm (excitation and emission wavelength). The fluorescence of FlOPs was presented as relative fluorescent intensity units per milliliter (FI/ml). The inter-assay and intra-assay CVs for FlOP measurement were 3.3 and 1.7%, respectively. We additionally tested the long-term stability of FlOPs among 16 participants in a pilot study. We found that FlOPs were stable at −80°C for at least 90 days. In the present study, all blood samples were collected between July and September 2019. Plasma FlOP levels were measured in October 2019. The storage duration of the blood samples and subsequent measurement of FlOP levels within 90 days in our study ensured the stability of FlOP levels.

### Anthropometric assessment

Body weight and height, without shoes and heavy clothes, were measured using a full automatic ultrasonic height and weight measuring instrument (SK-CK90, SONKA, Shenzhen Shuangjia Electronic Technology Co., Ltd., Guangdong, China). Body weight and height were recorded to the nearest 0.1 kg and 0.1 cm, respectively. BMI was calculated as weight (kg) divided by height (meters) squared. Participants were categorized as non-obese (BMI < 28 kg/m^2^) or obese (BMI ≥ 28 kg/m^2^) according to the cut-points recommended by the Working Group on Obesity in China (27).

### Ascertainment of other covariates

The covariates included in this study were socio-demographic data (e.g., age and sex), anthropometric variables (e.g., BMI), medical history (e.g., hypertension, type 2 diabetes mellitus status, and family history of kyphosis,), and lifestyle data (e.g., smoking and physical activity). Smoking was defined as the current or past use of tobacco. Frequent alcohol users were defined as a person who consumed an average of 3 or more units of alcohol per day. One unit is equivalent to half a pint (285 ml) of beer, one glass (125 ml) of wine, or a pub measure of spirits (8–10 g pure alcohol). Physical activity was computed using the frequency, duration, and intensity (light, moderate and heavy) of physical activity that was reported by participants and was subsequently expressed as metabolic equivalent hours per week (Met-hours/week) (28). Frequent users of calcium supplementation, dairy or soy products, and seafood were defined as an individual who ate these food items at least three times a week. Fasting blood glucose and blood pressure were obtained by physical examination. Diabetes mellitus was determined by either a fasting blood glucose ≥ 7.0 mmol/L, a self-reported diagnosis by a physician, or taking hypoglycaemic drugs (29). Hypertension was defined as a systolic blood pressure ≥ 140 mmHg or a diastolic blood pressure ≥ 90 mmHg, a self-reported diagnosis of hypertension by a physician, or the use of antihypertensive medication (30). Coronary heart disease, history of fracture, family history of osteoporosis diagnosis, and family history of kyphosis were self-reported.

### Statistical analysis

Descriptive data for the total population as well as by sex are provided. Continuous variables with a normal distribution are reported as means and standard deviations (SDs). Categorical variables are reported as frequencies and percentages. Data with a skewed distribution are presented as medians and interquartile ranges. The characteristics of participants by sex were compared using Student's *t*-test or Wilcoxon non-parametric test for continuous variables or Chi-square test for categorical variables. To test whether there was potential selection bias, we compared the baseline characteristics (i.e., age, sex, and BMI) between the included individuals for this study with the overall population from the 10 districts.

As the effect of oxidative stress on bone homeostasis is regulated by sex hormones and QUS measures were different between sexes (31, 32), all the regression analyses were performed stratified by sex. We used multivariable linear regression models to evaluate the associations of FlOPs with SOS and BUA. To better compare a person with a typical “high” value of FlOPs to a person with a typical “low” value, we rescaled the values of FlOP levels using the interquartile range, defined by the distance between the 25th and the 75th percentiles (33). FlOPs were further transformed into the natural logarithmic scale due to its skewed distribution. The following covariates were considered in all the adjustment models: age, BMI, smoking, frequent alcohol users, physical activity, frequent dairy or soy products, and history of fracture; sex and menopausal status were additionally adjusted for in the analysis of all the participants and in the analysis of only female participants, respectively. We included the covariates for adjustment if they were associated with either SOS or BUA at alpha = 0.1 in the bivariate analysis or were well-known risk factors for bone health. We also examined the relationships between covariates and FlOPs using bivariate analysis. Subgroup linear regression analyses by age (<60 years and ≥60 years), sex (male/female), BMI (<28 kg/m^2^ and ≥28 kg/m^2^), smoking (yes/no), frequent dairy or soy products use (yes/no), frequent alcohol use (yes/no), type 2 diabetes mellitus (yes/no), and hypertension (yes/no) were also performed. We conducted these subgroup analyses mainly because these stratified factors were related to FlOPs and/or QUS parameters (17, 32). To test whether there is interaction by these potential factors on FlOPs in relation to QUS parameters, we built the interaction terms (e.g., FlOPs^*^ age, FlOPs^*^sex, FlOPs^*^BMI, FlOPs^*^smoking, FlOPs^*^frequent dairy or soy products use, FlOPs^*^frequent alcohol use, FlOPs^*^type 2 diabetes mellitus, and FlOPs^*^hypertension) in the linear regression models. All analyses were conducted with SPSS (version 24.0, IBM SPSS Inc., Chicago, IL) or R (version: 4.0.0; R Foundation for Statistical Computing, Vienna, Austria) statistical software.

## Results

**Figure 1:**
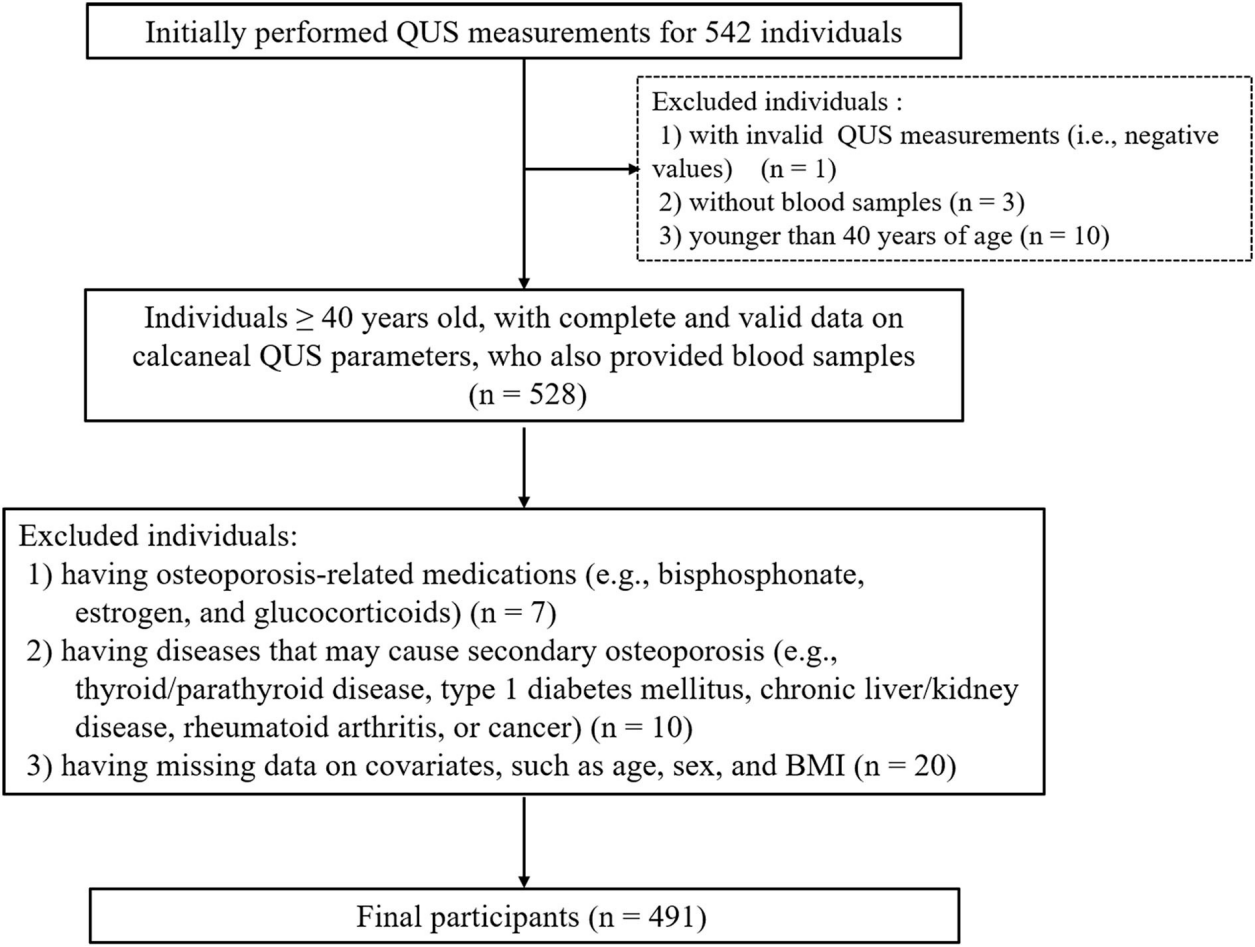
Inclusion and exclusion criteria of the participants.

We measured QUS for all 542 participants. However, we excluded one participant who had a negative BUA measurement value and further excluded ineligible individuals, resulting in a final study sample of 491 eligible subjects for analysis (Figure 1). Our participants were younger than the overall population from the 10 districts (65.2 vs.70.0 years; *P* < 0.05). However, there was no significant difference in the proportion of females (59.6 vs. 59.1%; *P* > 0.05) nor BMI (24.9 vs. 24.9 kg/m^2^) between the two populations. The characteristics of the included participants are shown in Table 1. The participants' median value of FlOPs was 143.0 FI/ml (IQR: 128.3–160.9 FI/ml; range: 97.6–478.8 FI/ml). Compared to males, females had a lower BMI and were less likely to be smokers and frequent alcohol users, and were more likely to consume seafood on a frequent basis (all *P* < 0.05). Females were also less likely to have type 2 diabetes mellitus, more likely to use calcium supplementation, and have a family history of kyphosis (all *P* < 0.05). Females also had a significantly lower median level of FlOPs compared to males (*P* < 0.05).

**Table 1:** Characteristics of the study participants.

**Variables**	**Total** **(*n* = 491)**	**Male** **(*n* = 201)**	**Female** **(*n* = 290)**
Age (years)	65.2 (9.7)	66.0 (9.1)	64.6 (10.1)
Body mass index (kg/m^2^)	24.9 (3.7)	**25.5 (3.5)**	**24.6 (3.7)**
Smoking (*n*, %)	82 (16.7%)	**74 (36.8%)**	**8 (2.8%)**
Frequent alcohol users (*n*, %)	52 (10.6%)	**50 (24.9%)**	**2 (0.7%)**
Physical activity (MET-hours/week)^a^	32.6 (2.3,44.1)	32.6 (7.0, 44.1)	32.6 (2.1, 44.1)
Use of calcium supplementation (*n*, %)	153 (31.2%)	**48 (23.9%)**	**105 (36.2%)**
Frequent dairy or soy products users (*n*, %)	210 (42.5%)	76 (37.8%)	134 (46.2%)
Frequent seafood users (*n*, %)	45 (9.2%)	**9 (4.5%)**	**36 (12.4%)**
Sunlight expose ≥ 30 min per day (*n*, %)	405 (82.5%)	167 (83.1%)	238 (82.1%)
Type 2 diabetes mellitus (*n*, %)	150 (30.6%)	**76 (37.8%)**	**74 (25.5%)**
Hypertension (*n*, %)	270 (55.0%)	112 (55.7%)	158 (54.5%)
Coronary heart disease (*n*, %)	82 (16.7%)	32 (15.9%)	50 (17.2%)
History of fracture (*n*, %)	24 (4.9%)	6 (3.0%)	18 (6.2%)
Family history of osteoporosis diagnosis (*n*, %)	36 (7.3%)	12 (6.0%)	24 (8.3%)
Family history of kyphosis (*n*, %)	38 (7.7%)	**8 (4.0%)**	**30 (10.3%)**
Menopause (*n*, %)	NA	NA	260 (89.7%)
FlOPs (FI/ml)^a^	143.0 (128.3, 160.9)	**148.1 (133.7, 165.4)**	**140.0 (124.9, 155.7)**
Natural logarithmic-scaled FlOPs (FI/ml)	5.00 (0.24)	**5.04 (0.24)**	**4.98 (0.24)**

Variables with a normal distribution are presented as mean (standard deviation);

a Variables with a skewed distribution are presented as median (interquartile range); categorical variables are presented as *n* (%). Bold-faced values indicate statistical significance at alpha = 0.05 between male and female. MET, metabolic equivalent Task; NA, Not applicable.

In the bivariate analyses, we observed significant positive associations between FlOPs with age, BMI, smoking, frequent alcohol use, type 2 diabetes mellitus, hypertension, and coronary heart disease; there was also a negative relationship between FlOPs and family history of kyphosis (all *P* < 0.05; Supplementary Table 1). As compared to males, females were associated with higher FlOPs. Hypertension and coronary heart disease were positively associated with FlOPs in males, whereas type 2 diabetes mellitus was positively associated with FlOPs in females (all *P* < 0.05).

In the bivariate analyses (Supplementary Tables 2, 3), SOS and BUA were inversely associated with age, while positively associated with frequent alcohol users. BMI had a positive relationship with SOS, but not with BUA. Frequent dairy or soy product users were only inversely associated with SOS. We only observed a positive association between smoking and BUA. Compared with males, females were associated with lower levels of SOS and BUA. In males, we only observed a statistically significant positive relationship between BMI and SOS. In females, age, coronary heart disease, and menopause were negatively associated with SOS, whereas age, hypertension, and menopause were negatively associated with BUA (all *P* < 0.05).

In the adjusted multivariable linear regression models (Table 2), we observed an inverse association between FlOPs and SOS (β for an increase of logarithmic interquartile range = −10.64; *P* = 0.018). Higher FlOP levels were marginally associated with lower SOS in females (β for an increase of logarithmic interquartile range = −9.68, *P* = 0.066), but not in males (β for an increase of logarithmic interquartile range = −11.84, *P* = 0.131). No significant association between FlOPs and BUA was noted (all *P* > 0.05; Table 2).

**Table 2:** Fluorescent oxidation products (FlOPs) and quantitative ultrasound (QUS) parameters: multivariable linear regression analysis.

**Subjects**	**FlOPs (FI/ml, 75th vs. 25th percentile)**	**SOS (m/s)**		**BUA (dB/mHz)**	
		β (95% CI)^*^	***P***	β (95% CI)^*^	***P***
Model 1^+^: total (*n* = 491)	160.9 vs. 128.3	−10.64 (−19.40, −1.88)	0.018	−0.14 (−2.75, 2.47)	0.893
Model 2^+^: male (*n* = 201)	165.4 vs. 133.7	−11.84 (−27.14, 3.47)	0.131	−1.59 (−6.61, 3.44)	0.537
Model 3^+:^ female (*n* = 209)	155.8 vs. 124.9	−9.68 (−19.95, 0.59)	0.066	0.83 (−1.88, 3.53)	0.548

* Per increase of logarithmic interquartile range in FlOPs.

^+^Model 1 adjusted for age, sex, BMI, smoking, frequent alcohol use, physical activity, frequent dairy or soy products use, and history of fracture. ^+^Model 2 adjusted for age, BMI, smoking, frequent alcohol use, physical activity, frequent dairy or soy products use, and history of fracture. ^+^Model 3 adjusted for factors in model 2 + menopausal status.

Subgroup analyses showed that the relationship between FlOPs and SOS was stronger in participants with type 2 diabetes mellitus (β = −24.700, *P* = 0.003) than in individuals without diabetes (β = −4.389, *P* = 0.405; *P* for interaction = 0.028; Table 3). The FlOP-SOS relationship was not modified by age, sex, BMI, smoking, frequent dairy or soy products, frequent alcohol users, or hypertension (all *P* for interaction > 0.05).

**Table 3:** Fluorescent oxidation products (FlOPs, Per increase of logarithmic interquartile range) and quantitative ultrasound (QUS) parameters: multivariable linear regression analysis stratified by age, sex, BMI, smoking, frequent alcohol users, frequent dairy or soy products users, type 2 diabetes mellitus, and hypertension^G^.

**Variables**	**Subgroup**	**Number**	**SOS (m/s)**			**BUA (dB/mHz)**			
			β	***P***	***P*** **for interaction**	β	***P***	***P*** **for interaction**
Age (year)	< 60	129	−4.760	0.590	0.489	1.335	0.612	0.842
	≥60	362	−12.676	0.016		−0.393	0.799	
Sex	Male^+^	201	−11.837	0.131	0.965	−1.585	0.537	0.566
	Female^= |^	290	−9.678	0.066		0.829	0.548	
BMI (kg/m^2^)	< 28	417	−7.541	0.093	0.150	0.117	0.928	0.756
	≥28	74	−24.931	0.126		−1.421	0.786	
Smoking	No	409	−10.231	0.026	0.388	0.216	0.877	0.880
	Yes	82	6.062	0.657		1.127	0.772	
Frequent alcohol users	No	439	−12.071	0.013	0.207	−0.478	0.736	0.294
	Yes	52	7.263	0.552		6.744	0.090	
Frequent dairy or soy products users	No	281	−13.216	0.045	0.603	−0.236	0.892	0.771
	Yes	210	−7.177	0.222		−0.231	0.912	
Type 2 diabetes mellitus	No	341	−4.389	0.405	0.028	0.286	0.849	0.637
	Yes	150	−24.700	0.003		−1.092	0.678	
Hypertension	No	221	−10.780	0.145	0.961	−0.134	0.943	0.985
	Yes	270	−11.447	0.051		−0.419	0.828	

^G^All models were adjusted for age, sex, BMI, smoking, frequent alcohol use, physical activity, frequent dairy or soy products use, and history of fracture, unless otherwise specified. ^+^Adjusted for age, BMI, smoking, frequent alcohol use, physical activity, frequent dairy or soy products use, and history of fracture. ^= |^Adjusted for age, BMI, smoking, frequent alcohol use, physical activity, frequent dairy or soy products use, history of fracture, and menopausal status.

## Discussion

In this community-based cross-sectional study, we observed that FlOPs were inversely associated with SOS in middle-aged and elderly adults. Higher FlOP levels were marginally associated with lower SOS in females, but not in males. No significant relationship between FlOPs and BUA was observed in the overall analysis nor by sex. The present findings expand our current knowledge of the relationship between FlOPs and QUS parameters.

As far as we know, this is the first study examining the relationship between FlOPs and QUS parameters. To some extent, our results were in agreement with two previous studies, in which higher FlOP levels are associated with lower BMD in male veterans (20) and an increased risk of hip fracture in postmenopausal women (21). Since QUS reflects the structural and mechanical properties of the bone (22, 23), the present study extended the evidence about the relationship between FlOPs and bone health in males and females. Moreover, among all the interaction terms, we observed that only type 2 diabetes mellitus status significantly modified the relationship between FlOPs and SOS. We did not find evidence to support smoking status as an effect modifier in the association between FlOPs and SOS. This is concordant with a previous study where smoking did not modify the relationship between FlOPs and BMD (20). Nevertheless, the possible explanation for the insignificant interaction terms between some subgroups (i.e., FlOPs^*^sex, FlOPs^*^smoking, and FlOPs^*^BMI) may be due to small sample size.

When compared our results with those of studies using other oxidative stress-related biomarkers, there are both consistencies and inconsistencies. For example, Basu et al. suggested that 8-iso-PGF_2α_, a lipid peroxidation marker, is negatively associated with SOS and BUA in Swedish adults (34). In the study of 868 Spanish men older than 50 years, Hernández et al. observed that higher levels of serum uric acid, a substance with antioxidant properties, are positively associated with all QUS parameters (14). In the present study, FlOPs were only associated with SOS, but not with BUA. This finding was partially consistent with the pooled results obtained by Enneman et al., which showed a statistically significant inverse association between homocysteine and SOS, but not BUA in older persons (15). Even though SOS and BUA are highly correlated (35), they reflect different aspects of bone properties and are influenced by various factors. SOS reflects the material property of the bone, such as the elastic modulus and compressive strength. BUA reflects bone microarchitecture and bone strength (23, 36). Previous *in vivo* study suggests that SOS has a stronger association with BMD than BUA as BUA failed to predict the mechanical properties of high-density trabecular bone (37). Further studies are warranted to elucidate this discrepancy.

Several potential mechanisms may explain the association between FlOPs and QUS parameters. Numerous lines of evidence suggest that oxidative stress is involved in the process of bone remodeling, inducing an imbalance between osteoclastic bone resorption and osteoblastic bone formation (7, 38, 39). Excessive ROS affects the differentiation and activity of osteoclasts by regulating mitogen-activated protein kinases (MAPKs), nuclear factor-kappa (NF-κB), and Ca^2+^-mediated signaling cascades (40). Baek et al. found that the number and activity of osteoclasts, as well as the receptor activator of nuclear factor-kappa B ligand (RANKL)/osteoprotegerin (OPG) ratio, were increased when hydrogen peroxide (H_2_O_2_) was added to human marrow mononuclear cells (41). Higher levels of oxidative stress decrease osteoprogenitor differentiation to the osteoblast cell lineage and promote the apoptosis of osteoblasts (7, 42). Bai et al. reported that H_2_O_2_-induced oxidative stress suppresses the osteoblastic differentiation process, manifested by a reduction of bone formation markers including alkaline phosphatase (ALP) (43).

Most of the existing studies focused on the relationship between oxidative stress and BMD (11, 12, 20). Bone strength is not only captured by BMD, but also by bone microarchitecture, bone mechanical properties, mineralization degree, and quality of collagens (44–46). In some situations, measurement of DXA is not available due to its high cost, ionizing radiation, and non-portability. QUS measurement of the calcaneus is a suitable method for screening osteoporosis. Moreover, compared to other oxidative stress-related biomarkers (i.e., MDA) that only reflect one specific aspect of oxidative damage (i.e., lipid peroxidation), FlOPs reflect the global level of oxidative damage *in vivo*. Overall, the present study provided supporting evidence for the association between FlOPs and bone mechanical and structural properties determined by QUS parameters. If our findings are confirmed in further studies, FlOPs may be a better biomarker for assessing the impact of global oxidative damage on BMD at the calcaneus and evaluating the effects of antioxidant use on bone health.

Several limitations of the present study need to be mentioned. First, due to the nature of the cross-sectional design, the temporal association between FlOPs and SOS cannot be determined. Second, our study had a small sample size; this may negatively impact the reliability of our results. Third, the possibility of residual confounding cannot be completely excluded, because some risk factors (i.e., vitamin D intake) were not included in the analysis. However, this limitation is likely to be minor as vitamin D intake is highly correlated with calcium intake (47, 48). Lastly, due to the difference in age between the included individuals selected from two urban districts in Changchun and the overall participants from the 10 districts, the present study may suffer from potential selection bias.

## Conclusions

In summary, plasma FlOP levels were inversely associated with SOS, but not with BUA in middle-aged and elderly adults. The present findings support the possibility of using FlOPs as a global biomarker to assess the impact of oxidative stress on the structural and mechanical properties of the bone. It would be worthwhile to conduct further studies to elucidate the roles of FlOPs in QUS parameters with a longitudinal study design.

## Data availability statement

The raw data supporting the conclusions of this article will be made available by the authors, without undue reservation.

## Ethics statement

The studies involving human participants were reviewed and approved by the Institutional Review Board (IRB) of China Medical University. The patients/participants provided their written informed consent to participate in this study.

## Author contributions

SY and YL designed the study. XS prepared the first draft of the paper. XS, QZ, BL, and YF contributed to the investigation and methodology of the present study. XS, MZ, and SY were responsible for the statistical analysis of the data. SY, MZ, AV, and HC reviewed and edited the manuscript. All authors read and approved the final manuscript.
