# Supplementary material for: Association between global biomarker of oxidative stress and quantitative ultrasound parameters in middle-aged and elderly adults: A cross-sectional study
Source: Front Public Health. 2023 Jan 6;10:1032550. doi: 10.3389/fpubh.2022.1032550 (PMC9853916; doi:10.3389/fpubh.2022.1032550)
Supplement: Supplementary file 1 [file Table_1.DOCX]

**Supplemental Table 1.** Participants’ characteristics and fluorescent oxidation products: bivariate analysis

| **Variables** | **Unit of Change* or Comparison** | **Total** | | **Male** | | **Female** | |
| --- | --- | --- | --- | --- | --- | --- | --- |
|  |  | **β** | ***P*** | **β** | ***P*** | **β** | ***P*** |
| Age (years) | 10.0 | 0.022 | 0.045 | 0.008 | 0.658 | 0.026 | 0.054 |
| Sex | Female vs. Male | 0.065 | 0.003 | NA | NA | NA | NA |
| Body mass index (kg/m^2^) | 4.0 | 0.022 | 0.041 | 0.013 | 0.446 | 0.022 | 0.117 |
| Smoking | Yes vs. No | 0.091 | 0.002 | 0.064 | 0.066 | 0.080 | 0.354 |
| Frequent alcohol use | Yes vs. No | 0.097 | 0.006 | 0.073 | 0.058 | -0.055 | 0.750 |
| **Physical activity (MET-hours/week)** |  |  |  |  |  |  |  |
| 22.0-44.0 | vs. < 22.0 | -0.010 | 0.702 | -0.034 | 0.413 | 0.002 | 0.943 |
| ≥ 44.0 | vs. < 22.0 | 0.004 | 0.867 | 0.011 | 0.789 | -0.003 | 0.927 |
| Use of calcium supplementation | Yes vs. No | -0.003 | 0.898 | -0.034 | 0.382 | 0.028 | 0.339 |
| Frequent dairy or soy products use | Yes vs. No | -0.001 | 0.992 | -0.005 | 0.897 | 0.012 | 0.684 |
| Frequent seafood use | Yes vs. No | 0.003 | 0.891 | 0.024 | 0.486 | -0.012 | 0.691 |
| Sunlight expose ≥ 30 minutes per day | Yes vs. No | -0.007 | 0.819 | -0.052 | 0.242 | 0.022 | 0.556 |
| Type 2 diabetes mellitus | Yes vs. No | 0.068 | 0.004 | 0.032 | 0.355 | 0.085 | 0.009 |
| Hypertension | Yes vs. No | 0.055 | 0.011 | 0.069 | 0.040 | 0.045 | 0.116 |
| Coronary heart disease | Yes vs. No | 0.069 | 0.019 | 0.126 | 0.006 | 0.034 | 0.369 |
| History of fracture | Yes vs. No | -0.064 | 0.206 | -0.031 | 0.751 | -0.069 | 0.302 |
| Family history of osteoporosis diagnosis | Yes vs. No | -0.030 | 0.467 | -0.004 | 0.953 | -0.036 | 0.487 |
| Family history of kyphosis | Yes vs. No | -0.086 | 0.034 | -0.083 | 0.333 | -0.070 | 0.131 |
| Menopausal status | Yes vs. No | NA | NA | NA | NA | 0.040 | 0.396 |

*Approximately 1 SD. MET: metabolic equivalent Task. NA: Not applicable.

**Supplemental Table 2.** Participants’ characteristics and speed of sound (SOS): bivariate analysis

| **Variables** | **Unit of Change* or**  **Comparison** | **Total** | | **Male** | | **Female** | |
| --- | --- | --- | --- | --- | --- | --- | --- |
|  |  | **β** | ***P*** | **β** | ***P*** | **β** | ***P*** |
| Age (years) | 10.0 | -6.410 | <0.001 | -3.888 | 0.052 | -8.086 | < 0.001 |
| Sex | Female vs. Male | -4.654 | 0.038 | NA | NA | NA | NA |
| Body mass index (kg/m^2^) | 4.0 | 2.449 | 0.026 | 4.223 | 0.028 | 0.927 | 0.486 |
| Smoking | Yes vs. No | 3.692 | 0.212 | 1.320 | 0.733 | 0.291 | 0.972 |
| Frequent alcohol use | Yes vs. No | 7.327 | 0.041 | 5.661 | 0.188 | -2.836 | 0.862 |
| **Physical activity (MET-hours/week)** |  |  |  |  |  |  |  |
| ≥ 22.1; < 44.1 | vs. < 22.1 | 0.546 | 0.843 | 7.236 | 0.122 | -4.395 | 0.191 |
| ≥ 44.1 | vs. < 22.1 | 1.055 | 0.694 | 3.420 | 0.454 | -0.582 | 0.857 |
| Use of calcium supplementation | Yes vs. No | -4.641 | 0.051 | -2.253 | 0.606 | -5.052 | 0.070 |
| Frequent dairy or soy products use | Yes vs. No | -7.186 | 0.018 | -3.642 | 0.343 | 3.707 | 0.169 |
| Frequent seafood use | Yes vs. No | 2.052 | 0.362 | -0.770 | 0.840 | 3.989 | 0.146 |
| Sunlight expose ≥ 30 minutes per day | Yes vs. No | -1.569 | 0.589 | 3.357 | 0.499 | -4.961 | 0.156 |
| Type 2 diabetes mellitus | Yes vs. No | 2.129 | 0.374 | 1.373 | 0.721 | 1.614 | 0.601 |
| Hypertension | Yes vs. No | -1.779 | 0.422 | 1.922 | 0.608 | -4.427 | 0.101 |
| Coronary heart disease | Yes vs. No | -3.650 | 0.217 | 2.041 | 0.689 | -7.176 | 0.043 |
| History of fracture | Yes vs. No | -8.636 | 0.091 | -20.500 | 0.060 | -3.554 | 0.524 |
| Family history of osteoporosis diagnosis | Yes vs. No | 2.344 | 0.580 | 1.067 | 0.892 | 3.584 | 0.463 |
| Family history of kyphosis | Yes vs. No | 1.944 | 0.638 | -3.472 | 0.716 | 4.833 | 0.273 |
| Menopausal status | Yes vs. No | NA | NA | NA | NA | -23.635 | < 0.001 |

*Approximately 1 SD.

MET: metabolic equivalent Task. NA: Not applicable.

**Supplemental Table 3.** Participants’ characteristics and broadband ultrasound attenuation (BUA): bivariate analysis

| **Variables** | **Unit of Change* or**  **Comparison** | **Total** | | **Male** | | **Female** | |
| --- | --- | --- | --- | --- | --- | --- | --- |
|  |  | **β** | ***P*** | **β** | ***P*** | **β** | ***P*** |
| Age (years) | 10.0 | -1.178 | 0.003 | 0.152 | 0.812 | -2.112 | < 0.001 |
| Sex | Female vs. Male | -3.697 | < 0.001 | NA | NA | NA | NA |
| Body mass index (kg/m^2^) | 4.0 | 0.223 | 0.497 | -0.165 | 0.789 | 0.107 | 0.758 |
| Smoking | Yes vs. No | 2.126 | 0.016 | 0.104 | 0.933 | -1.167 | 0.584 |
| Frequent alcohol use | Yes vs. No | 3.221 | 0.003 | 1.060 | 0.441 | 1.916 | 0.650 |
| **Physical activity (MET-hours/week)** |  |  |  |  |  |  |  |
| ≥ 22.1; < 44.1 | vs. < 22.1 | 1.297 | 0.114 | 2.010 | 0.177 | 0.581 | 0.506 |
| ≥ 44.1 | vs. < 22.1 | 1.405 | 0.078 | 2.053 | 0.160 | 0.837 | 0.321 |
| Use of calcium supplementation | Yes vs. No | -0.271 | 0.703 | 0.816 | 0.559 | -0.063 | 0.930 |
| Frequent dairy or soy products use | Yes vs. No | -0.291 | 0.749 | -1.078 | 0.380 | 1.234 | 0.077 |
| Frequent seafood use | Yes vs. No | 0.074 | 0.913 | -0.639 | 0.599 | 0.554 | 0.437 |
| Sunlight expose ≥ 30 minutes per day | Yes vs. No | 0.640 | 0.459 | 2.761 | 0.081 | -0.868 | 0.340 |
| Type 2 diabetes mellitus | Yes vs. No | -1.039 | 0.145 | -2.140 | 0.080 | -1.108 | 0.166 |
| Hypertension | Yes vs. No | -0.905 | 0.171 | -0.163 | 0.892 | -1.491 | 0.033 |
| Coronary heart disease | Yes vs. No | -0.688 | 0.435 | -0.048 | 0.976 | -0.963 | 0.297 |
| History of fracture | Yes vs. No | -2.526 | 0.097 | -5.577 | 0.110 | -0.655 | 0.651 |
| Family history of osteoporosis diagnosis | Yes vs. No | 0.480 | 0.703 | -1.411 | 0.574 | 1.911 | 0.131 |
| Family history of kyphosis | Yes vs. No | -1.934 | 0.116 | 1.100 | 0.718 | -1.796 | 0.117 |
| Menopausal status | Yes vs. No | NA | NA | NA | NA | -5.130 | < 0.001 |

*Approximately 1 SD.

METs: metabolic equivalent hours. NA: Not applicable.
